# CLAG±DAC方案治疗难治复发性急性髓系白血病的临床研究

**DOI:** 10.3760/cma.j.cn121090-20240604-00203

**Published:** 2024-09

**Authors:** 雯玺 华, 卫芹 姚, 萌 周, 嘉乾 戚, 慧珠 康, 如菊 王, 成森 蔡, 跃均 刘, 德沛 吴, 悦 韩

**Affiliations:** 苏州大学附属第一医院，江苏省血液研究所，国家卫生健康委员会血栓及止血重点研究室，国家血液系统疾病临床医学研究中心，苏州 215006 The First Affiliated Hospital of Soochow University, Jiangsu Institute of Hematology, Key Laboratory of Thrombosis and Hemostasis of Ministry of Health, National Clinical Research Center for Hematologic Diseases, Suzhou 215006, China

**Keywords:** 白血病，髓系，急性, 复发, 难治, 抗肿瘤联合化疗方案, 地西他滨, FLT3-ITD突变, Leukemia, myeloid, acute, Relapsed, Refractory, Anti-tumor combination chemotherapy regimen, Decitabine, FLT3-ITD mutant

## Abstract

**目的:**

探讨CLAG（克拉屈滨、阿糖胞苷、G-CSF）±地西他滨（DAC）方案对难治复发性急性髓系白血病（R/R AML）的疗效及其影响因素。

**方法:**

回顾性分析2017年1月至2021年12月苏州大学附属第一医院收治的应用CLAG+DAC或单纯CLAG方案治疗的R/R AML病例，收集患者的基本特征、个体化治疗方案、治疗反应、疾病进展和生存状态。分析影响CLAG±DAC化疗方案疗效的因素，并采用Kaplan-Meier方法计算总生存（OS）期。

**结果:**

共纳入53例患者，男性患者33例，患者平均年龄为40.6岁。33例患者在CLAG±DAC方案化疗1个疗程后达完全缓解（CR）或CR伴血细胞不完全恢复，6例获部分缓解，14例未缓解。有32例患者最终行造血干细胞移植。至随访截止，患者中位OS期为55.9个月。应用CLAG±DAC方案化疗后疾病缓解的患者OS期明显长于未缓解的患者（*P*<0.001）。多因素分析结果显示，联合DAC（*OR*＝4.60，95％*CI* 1.14～23.50, *P*＝0.04）、合并DNMT3A突变（*OR*＝0.14，95％*CI* 0.01～0.89, *P*＝0.05）是CLAG±DAC化疗方案疗效的影响因素。R/R AML合并FLT3-ITD突变患者应用CLAG+DAC方案缓解率相对更高（*OR*＝10.84，95％*CI* 1.48～288.50, *P*＝0.04）。

**结论:**

CLAG±DAC方案对R/R AML患者疗效显著，且DAC联合CLAG方案更适用于合并FLT3-ITD突变的R/R AML患者。

## Abstract (en)

**Objective:**

To investigate the efficacy and prognosis of CLAG±DAC（Clofarabine, Cytarabine, G-CSF±Decitabine）chemotherapy in patients with relapsed/refractory acute myeloid leukemia（R/R AML）.

**Methods:**

Continuous cases of R/R AML treated with the CLAG+DAC protocol or CLAG alone at the First Affiliated Hospital of Soochow University from January 2017 to December 2021 were retrospectively analyzed. The baseline characteristics, individual treatment regimen, treatment effect, disease progression, and survival status of patients were recorded. The factors influencing the efficacy of the CLAG±DAC chemotherapy regimens were analyzed, and the overall survival（OS）time after reinduction was calculated using the Kaplan-Meier method.

**Results:**

This study included a total of 53 patients, with 33 male patients and an average age of 40.6 years. Thirty-three patients achieved complete remission（CR+CRi）of the disease after the CLAG±DAC chemotherapy regimen and six patients achieved partial remission（PR）, while 14 did not. Thirty-two patients eventually underwent hematopoietic stem cell transplantation, and the median OS of the patients was 55.9 months until follow-up. Patients with disease remission after the application of the CLAG±DAC chemotherapy had a significantly longer survival time than those without remission（*P*<0.001）. The results of the multifactorial analysis have revealed that combined DAC（*OR*＝4.60, 95% *CI* 1.14–23.5, *P*＝0.04）and DNMT3A mutation（*OR*＝0.14, 95% *CI* 0.01–0.89, *P*＝0.05）were the factors influencing the efficacy of the CLAG±DAC chemotherapy regimen. The remission rate was relatively higher in patients with R/R AML combined with FLT3-ITD mutation by applying the DAC+CLAG regimen（*OR*＝10.84, 95%*CI* 1.48–288.50, *P*＝0.04）.

**Conclusion:**

The CLAG±DAC regimen is considered effective in patients with R/R AML, whereas decitabine combined with the CLAG regimen is more suitable for patients with R/R AML combined with FLT3-ITD mutation.

目前对于难治复发性急性髓系白血病（R/R AML）临床上并没有标准的治疗方案，许多挽救性强化疗方案已被证明有良好的抗白血病效应，包括FLAG（氟达拉滨、阿糖胞苷、G-CSF）方案、MEC（米托蒽醌、依托泊苷、阿糖胞苷）方案、大剂量阿糖胞苷、ADE（阿糖胞苷、柔红霉素、依托泊苷）方案等，其临床有效率在40％～55％[1]–[5]。基于国内外许多临床中心报道CLAG（克拉屈滨、阿糖胞苷、G-CSF）方案诱导治疗R/R AML疗效显著，国内外已经将CLAG方案作为R/R AML的推荐疗法，但仍有部分患者对CLAG方案不敏感[6]–[7]。地西他滨（DAC）作为去甲基化药物，在肿瘤治疗中举足轻重，目前国内外尚未有CLAG±DAC方案治疗R/R AML疗效的报道。因此本研究回顾性分析了我院2017至2022年使用CLAG±DAC化疗方案的R/R AML患者的临床资料及生存状况，并分析疗效影响因素。

## 病例与方法

1. 病例资料：选取于2017年1月至2021年12月在苏州大学附属第一医院治疗的53例R/R AML患者。复发的定义：完全缓解（CR）后外周血再次出现白血病细胞或骨髓原始细胞>5％（除外巩固化疗后的骨髓重建等原因）；或髓外出现白血病细胞浸润。难治的定义：经过2个疗程标准方案治疗后未获得CR的初治病例；第1次CR后经过巩固强化治疗，12个月内复发者；12个月后复发但经过常规化疗方案治疗不能缓解者；2次或多次复发者；髓外白血病持续存在者[8]。所有患者均采用CLAG或CLAG+DAC的方案再诱导化疗。收集患者的年龄、性别、住院时间、初诊白细胞计数、骨髓细胞形态、免疫表型、细胞遗传学和分子遗传学、基因突变、既往化疗疗程数、疾病缓解情况、是否移植、存活状态和生存时间等临床资料。

2. 治疗方案：治疗方案的选择通过结合患者目前R/R AML的病情现状和临床上的用药经验决定，用药方案已取得患者与家属的知情同意。化疗方案的具体用药如下：单纯CLAG方案：克拉屈滨5 mg/m^2^，第1～5天；阿糖胞苷1～2 g/m^2^，克拉屈滨用后4 h使用，第1～5天，静脉滴注3 h；G-CSF 300 µg/m^2^，第0～5天，WBC>20×10^9^/L暂停。DAC+CLAG：DAC 20 mg/m^2^，第1～3天，CLAG方案同前。

3. 疗效及不良反应评价：根据2022年欧洲白血病网（ELN）指南[9]，疗效定义如下，CR：骨髓原始细胞<5％，外周血未见原始细胞，无髓外病变，中性粒细胞绝对计数≥1.0×10^9^/L且PLT≥100×10^9^/L；CR伴血细胞不完全恢复（CRi）：骨髓原始细胞<5％，外周血未见原始细胞，无髓外病变，中性粒细胞绝对计数<1.0×10^9^/L或者PLT<100×10^9^/L；部分缓解（PR）：血细胞恢复达CR，骨髓原始细胞比例5％～25％，且较治疗前下降至少50％。未缓解（NR）：评估不符合CR、CRi、PR。总生存（OS）期定义为开始使用CLAG±DAC方案至患者死亡或随访截止时间。不良反应评价依据美国国家癌症研究所常见不良反应事件评价标准[10]进行评价。

4. 随访：主要通过门诊就诊以及电话的形式进行随访。随访的内容包括病情评估、疾病进展、复发或者死亡。末次随访时间为2024年3月1日。

5. 统计学处理：采用统计学软件R 4.2.0进行统计分析，对于计量资料符合正态分布的用均数±标准差表示，不符合正态分布的用*M*（范围）表示。计数资料采用频率表示，组间的差异比较采用卡方检验。应用Logistic回归模型进行单因素和多因素分析来评估影响CR的相关因素，单因素分析纳入CLAG类型、性别、基因突变、疗程数，单因素中*P*<0.1的纳入多因素分析，以*P*<0.05为差异有统计学意义。采用Kaplan-Meier法描绘生存曲线，采用Log-rank检验比较生存差异。

## 结果

1. 一般资料：53例患者中女20例，男33例，平均年龄为40.6岁。复发32例，难治21例。其中有3例患者为骨髓增生异常综合征转化的AML，1例为真性红细胞增多症继发AML，其余49例患者均为原发AML。遵循ELN指南标准基于患者骨髓细胞遗传学与分子遗传学情况进行疾病危险分层，其中低危组7例、中危组18例、高危组28例。初诊时中位WBC为25.3（1.0～362.2）×10^9^/L。在使用CLAG±DAC方案之前，所有患者都经历过1～6个疗程的化疗。所有患者均合并有不同种类的基因突变，52.8％的患者伴有FLT3-ITD突变。化疗方案中34例患者单纯采用CLAG方案，19例患者采用CLAG+DAC方案。最终有32例患者进行了造血干细胞移植（表1）。

**表1:** CLAG±DAC化疗方案治疗53例难治复发急性髓系白血病（AML）患者的基本临床特征

临床特征	患者总体（53例）	CLAG组（34例）	CLAG+DAC组（19例）	统计量	*P*值
年龄（岁，*x*±*s*）	40.6±13.7	42.1±14.3	37.7±12.6	*t*＝1.12	0.27
性别（例，男/女）	33/20	21/13	12/7	*χ*^2^＝0.01	0.92
AML类型［例（%）］				*χ*^2^＝0.36	0.61
原发	49（92.4）	32（94.1）	17（89.5）		
继发	4（7.6）	2（5.9）	2（10.5）		
疾病状态［例（%）］				*χ*^2^＝2.19	0.14
复发	32（60.4）	18（52.9）	14（73.7）		
难治	21（39.6）	16（47.1）	5（26.3）		
移植［例（%）］				*χ*^2^<0.01	0.99
否	13/45（28.9）	9/30（30.0）	4/15（26.7）		
是	21/45（71.1）	21/30（70.0）	11/15（73.3）		
既往化疗疗程数［例（%）］				*χ*^2^＝5.93	0.33
1	32（60.4）	17（50.0）	15（78.9）		
2	9（17.0）	7（20.6）	2（10.5）		
3	5（9.4）	4（11.8）	1（5.3）		
4	4（7.5）	4（11.8）	0（0）		
5	1（1.9）	1（2.9）	0（0）		
6	2（3.8）	1（2.9）	1（5.3）		
WBC［×10^9^/L，*M*(范围)］	25.3（1.0～362.2）	23.2（1.0～272.0）	27.0（2.0～362.2）	*z*＝−1.01	0.32
原始幼稚细胞（%，*x*±*s*）	62.8±22.1^a^	64.1±21.3	60.4±24.0	*t*＝0.55	0.59
危险分层［例（%）］				*χ*^2^＝4.63	0.13
低危	7（13.2）	5（14.7）	2（10.5）		
中危	18（34.0）	8（23.5）	10（52.6）		
高危	28（52.8）	21（61.8）	7（36.8）		
伴FLT3-ITD突变［例（%）］				*χ*^2^＝2.89	0.09
是	28（52.8）	15（44.1）	13（68.4）		
否	25（47.2）	19（55.9）	6（31.6）		
疗效［例（%）］				*χ*^2^＝0.84	0.87
CR+CRi	33（62.3）	20（58.8）	13（68.4）		
PR	6（11.3）	4（11.8）	2（10.5）		
NR	14（26.4）	10（29.4）	4（21.1）		

**注** CLAG：克拉屈滨、阿糖胞苷、G-CSF；DAC：地西他滨；^a^有4例患者原始幼稚细胞数缺失。CR：完全缓解；CRi：CR伴血细胞不完全恢复；PR：部分缓解；NR：未缓解

2. 疗效分析：53例患者均在我中心进行再诱导化疗，1个疗程后有62.3％的患者CR。共33例患者在使用CLAG±DAC 1个疗程后达到了CR+CRi，6例患者达到PR，14例NR。单因素分析显示联合DAC、DNMT3A突变、C-KIT突变、ASXL1突变是影响CLAG±DAC化疗方案疗效的因素。将单因素*P*<0.1的因素纳入多因素分析，结果提示未联合DAC、伴有DNMT3A突变为影响应用含有CLAG方案疗效的独立危险因素（表2）。53例患者中有28例合并FLT3-ITD的突变，分析两种化疗方案对R/R AML合并FLT3-ITD突变患者的治疗疗效，结果提示CLAG+DAC较单纯CLAG方案疗效更好（*OR*＝10.84，95％*CI* 1.48～288.50，*P*＝0.04），另外FLT3-ITD合并DNMT3A突变的患者使用含CLAG化疗方案的疗效较差（*OR*＝0.04，95％*CI* 0.001～0.49，*P*＝0.03）（表3）。

**表2:** 影响CLAG±DAC方案治疗难治复发性急性髓系白血病患者1个疗程疗效的多因素分析

变量	*OR*值（95%*CI*）	*P*值
CLAG类型		
CLAG	1	–
CLAG+DAC	4.60 (1.14~23.50)	0.04
DNMT3A突变		
是	0.14 (0.01~0.89)	0.05
否	1	–
C-KIT突变		
是	3.08 (0.37~66.40)	0.35
否	1	–
ASXL1突变		
是	0.65 (0.12~3.52)	0.61
否	1	–

**注** CLAG：克拉屈滨、阿糖胞苷、G-CSF；DAC：地西他滨

**表3:** 影响CLAG±DAC方案治疗合并FLT3-ITD突变难治复发性急性髓系白血病患者1个疗程疗效的多因素分析

变量	OR值（95%*CI*）	*P*值
CLAG类型		
CLAG	1	–
CLAG+DAC	10.84(1.48~288.50)	0.04
性别		
男	1	–
女	1.15(0.17~8.67)	0.89
DNMT3A突变		
是	0.04(0.001~0.49)	0.03
否	1	–

**注** CLAG：克拉屈滨、阿糖胞苷、G-CSF；DAC：地西他滨

3. 生存分析：截至2024年3月1日，25例患者死亡，28例存活，所有患者中位OS时间为55.9个月。应用含CLAG化疗方案后疾病缓解的患者OS明显优于CLAG后未缓解的患者（*P*<0.001）（图1）。比较CLAG+DAC方案组和单纯CLAG方案组患者生存率，差异无统计学意义（*P*＝0.830）（图2）。

**图1:**
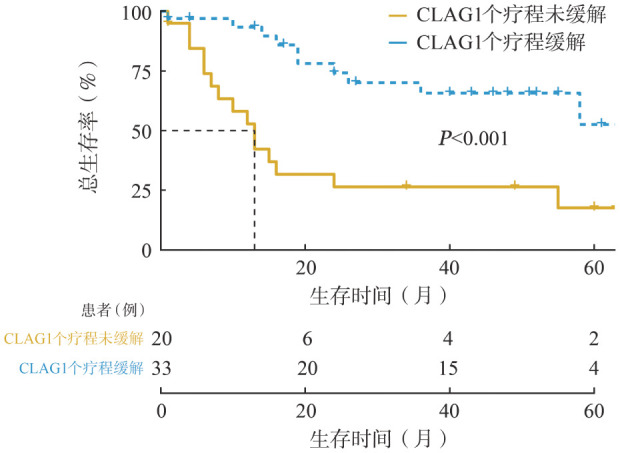
CLAG±DAC方案诱导治疗1个疗程疾病缓解状态对难治复发性急性髓系白血病总生存的影响 **注** CLAG：克拉屈滨、阿糖胞苷、G-CSF；DAC：地西他滨

**图2:**
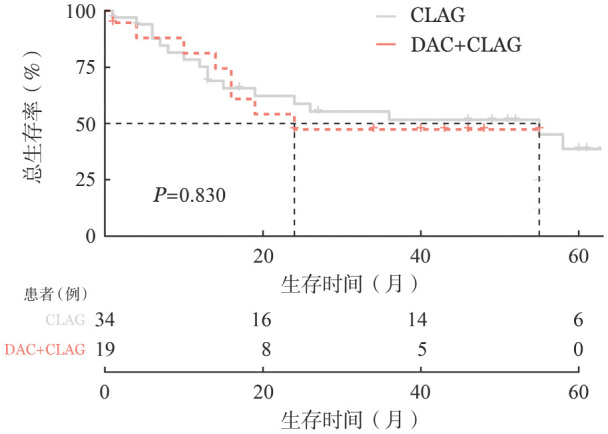
CLAG+DAC方案和单纯CLAG方案治疗复发难治急性髓系白血病患者总生存的比较 **注** CLAG：克拉屈滨、阿糖胞苷、G-CSF；DAC：地西他滨

4. 不良反应：本研究中所有患者在使用CLAG±DAC化疗方案后均出现了4级骨髓抑制，42例患者化疗期间出现发热，其中肺部感染18例，败血症12例，9例患者发生皮肤黏膜软组织感染，3例患者出现肝酶升高，经保肝治疗后好转，1例患者出现真菌性败血症、肺泡出血、凝血功能异常，抗感染、止血和改善凝血功能治疗无效后死亡。其他不良反应包括腹泻、恶心、呕吐，对症治疗后均好转，无患者出现肾功能损伤。单纯CLAG方案组的中性粒细胞恢复（中性粒细胞绝对计数>0.5×10^9^/L）中位时间14（7～25）d，血小板恢复（PLT>20×10^9^/L）中位时间22（11～34）d，CLAG+DAC方案组中性粒细胞恢复中位时间14（10～24）d，血小板恢复中位时间23（11～36）d。

## 讨论

AML是所有白血病中最常见的类型，其分子表型、遗传特征和临床预后都存在异质性，患者个体间的差异使得部分患者对经典化疗方案不敏感。有研究报道，约有40％的年轻患者对AML的诱导治疗无效，在老年以及高危患者中所占比例更大，对这部分患者，再诱导后行异基因造血干细胞移植是延长生存时间的最好办法，尽管如此，他们复发风险依旧较高[11]。

2000年Robak等[12]首次报道用以CLAG方案治疗R/R AML患者，再诱导后有50％的患者达到CR，且所有患者可耐受不良反应。既往有大量研究比较CLAG方案和MEC方案以及CLAG和FLAG方案的疗效，研究结果证实CLAG方案组患者中位无进展生存期和OS期明显长于MEC组，而CLAG和FLAG方案组间无明显疗效差异[13]–[14]。既往有研究数据显示DAC联合CAG方案治疗R/R AML的CR率为55％，1年OS率为70％[15]。另有研究发现DAC联合吉姆单抗或DAC联合DAG方案在R/R AML也有较好的疗效[16]–[17]。DAC是一种核苷抑制剂，通过磷酸化成5-AZA-dCTP后与DNA结合，以此抑制DNMT导致低甲基化和基因表达改变，在MDS和AML的治疗中发挥作用；另外它还可能引起DNA合成和复制异常，从而降低细胞增殖，诱导细胞凋亡[18]。研究表明低剂量DAC不仅有去甲基化作用，还能够使得沉默基因恢复表达；而高浓度DAC主要发挥细胞毒性作用[19]。目前国内外各中心报道CLAG治疗后的CR率在40％～60％[20]–[23]，但目前国内外缺少DAC联合CLAG对R/R AML的治疗评估。我院纳入53例R/R AML患者，有34例患者单纯使用CLAG化疗方案，19例联合DAC，总体CR率达到62.3％。DAC联合CLAG化疗方案后患者的缓解率较单纯用CLAG方案高（*P*<0.05）。另外，我们研究发现在FLT3-ITD突变的R/R AML患者中，CLAG+DAC方案化疗后缓解率明显高于单纯使用CLAG组，两组间差异有统计学意义。因此我们认为对于R/R AML合并FLT3-ITD突变的患者，更推荐使用DAC+CLAG方案再诱导化疗。

另外本项研究中有7例患者伴随DNMT3A突变，该基因发生突变的患者只有28.6％达到CR，且其中6例最终死亡，预后较差，伴随DNMT3A突变的患者中有86％合并FLT3-ITD突变，且NPM1和IDH2的伴随突变概率也较高。我们的研究结果提示CLAG±DAC方案不利于DNMT3A突变的患者，由于我们的样本量有限，需要更多的临床数据进一步验证。既往研究证实DNMT3A突变诱导造血干细胞扩增，与FLT3-ITD和NPM1c协同在体内诱导AML，促进对蒽环类药物的耐药[24]。前人研究也显示AML患者中DNMT3A突变往往伴随着FLT3、NPM1、IDH1和IDH2的突变，且这部分患者OS更差[25]。

本研究结果显示是否联合DAC对患者OS期无明显影响，其原因可能是只有不到一半的患者在行CLAG+DAC化疗后桥接造血干细胞移植，而单纯使用CLAG组有87％的患者最终行造血干细胞移植。本研究为单中心回顾性研究，样本量有限，同时缺少接受其他化疗方案的对照组，因此结论需要更大样本的前瞻性临床研究来验证。

总之，CLAG±DAC化疗方案对R/R AML有较好疗效，患者总体耐受性良好，且DAC联合CLAG方案较单纯CLAG方案效果更好，合并FLT3-ITD突变的R/R AML更适合用CLAG+DAC方案。另外，再诱导后本病仍未缓解的患者，即使后续行造血干细胞移植，总体预后仍较差。
